# COVID-19 Infection and Response to Vaccination in Chronic Kidney Disease and Renal Transplantation: A Brief Presentation

**DOI:** 10.3390/life12091358

**Published:** 2022-08-31

**Authors:** Stamatia Stai, Georgios Lioulios, Michalis Christodoulou, Efstratios Kasimatis, Asimina Fylaktou, Maria Stangou

**Affiliations:** 1 Department of Nephrology Aristotle, University of Thessaloniki, Hippokration Hospital, 54642 Thessaloniki, Greece; 2 Department of Immunology, National Peripheral Histocompatibility Center, Hippokration Hospital, 54642 Thessaloniki, Greece

**Keywords:** chronic kidney disease, renal transplantation, immune system, COVID-19, vaccination

## Abstract

Chronic kidney disease (CKD) is associated with phenotypic and functional changes in the immune system, followed by detrimental clinical consequences, such as severe infections and defective response to vaccination. Two years of the pandemic, due to severe acute respiratory syndrome coronavirus 2 (SARS-CoV-2), have undoubtedly changed the world; however, all efforts to confront infection and provide new generation vaccines tremendously improved our understanding of the mechanisms of the immune response against infections and after vaccination. Humoral and cellular responses to vaccines, including mRNA vaccines, are apparently affected in CKD patients, as elimination of recent thymic emigrant and naïve lymphocytes and regulatory T-cells, together with contraction of T-cell repertoire and homeostatic proliferation rate, which characterized CKD patients are responsible for impaired immune activation. Successful renal transplantation will restore some of these changes, although several epigenetic changes are irreversible and even accelerated by the induction of immunosuppression. Response to vaccination is definitely impaired among both CKD and RT patients. In the present review, we analyzed the differences in immune response after vaccination between these patients and healthy individuals and depicted specific parameters, such as alterations in the immune system, predisposing to this deficient response.

## 1. COVID-19 Infection in CKD and RT Patients

COVID-19 infection has been characterized by rapid widespread all over the world and the high mortality rates among infected individuals. Certain populations, for various reasons, showed not only an increased incidence of the disease but also increased morbidity and mortality. The presence of chronic kidney disease, especially end-stage kidney disease and hemodialysis, was one of the main factors, very soon recognized, after the pandemic outbreak, as risk factors predisposing to highly contagiousness and adverse outcome of the disease. The absolute cumulative incidence of SARS-CoV-2 infection in ESKD patients undergoing hemodialysis was almost 4-fold higher compared with a healthy population of similar ethnicity, sex, and age. However, even more devastating are the results of mortality, with hemodialysis patients experiencing extremely high mortality rates due to COVID-19, reaching almost 30% and 44% in ages < 75 and >75 years old, respectively, 5.5- and 11-fold higher than the general population, respectively [1,2]. Data were only slightly better after kidney transplantation. Based on the European Renal Association COVID-19 Database (ERACODA) database, which analyzed data from infected RT and dialysis patients, mortality rates during the first month were almost identical, 21% and 25%, respectively, correlated with patients’ age and frailty. Very importantly, this study showed a significant increase in mortality among recently transplanted patients (<1 year), probably due to immunosuppression treatment [3].

However, all the above studies were contacted at pre-vaccine times, when physicians were not familiar with disease transmission and outcome, but most of all, there were no available mechanisms to prevent or defend against infection. Anti-SARS-CoV-2 vaccination has attracted the main physicians’ and patients’ interest and hope for a substantial weapon against disease. Response to vaccination is therefore of major importance, particularly in those patients with increased susceptibility to transmission and increased incidence of morbidity and mortality.

## 2. Understanding mRNA Vaccine-Induced Immune Response

The development of vaccines has undoubtedly been a crucial step toward the advancement and optimization of public health, especially since the proper establishment of relevant national programs promoting their wide application. The essence of their use lies in their ability to cause artificial exposure to (a) specific antigen(s) (Ag) of pathogens, thus inducing an immune response that aims to prevent future infection or decrease the severity of disease manifestations. There are various types of vaccines, including live attenuated, killed whole organism, toxoid, subunit, containing virus-like particles, protein-polysaccharide conjugate, outer membrane vesicle (OMV)-based, nucleic acid (specifically DNA—as plasmids—and messenger RNA—mRNA)-based, viral and bacterial vectored, as well as antigen-presenting cell (APC)-targeting, with the two latter being at an experimental stage of development [4,5]. 

Although vaccines have generally been thought to act by mobilizing B-cells in order to produce an effective humoral response, it is now known that T-cells are also actuated, even though their exact role has not been fully clarified. After interacting with foreign-Ag-presenting dendritic cells (DCs) through their T-cell receptor (TCR), activated cluster of differentiation (CD)4+ T-cells mediate B-cell development in the lymph nodes and promote the subsequent maturation of antibody (Ab) response and the creation of short-lived plasma cells (PCs), responsible for the rapid release of Ag-specific Abs. In the process, memory B-cells and long-lived PCs are also produced. As for T-cells, the difficulty to shed light on their precise function results (to some degree) from reduced accessibility to a considerable percentage of them (e.g., those residing in tissues), which renders calculations regarding alterations in their numbers harder [4]. It is not surprising that several authors have supported that the utilization of adjuvants that selectively augment T-cell responses is a promising step toward the achievement of more effective and enduring immunity induction [6].

mRNA vaccines are a propitious alternative to conventional approaches, as they offer a satisfactory combination of efficacy and safety (they do not pose a risk of infection or integration of the vector into the host cell DNA, while mRNA can be degraded by normal cellular processes), as well as the potential for rapid, large-scale production [7,8]. Recent research has been focusing on two basic types of mRNA vaccines, the non-replicating and the self-amplifying ones (saRNA). Their structure mimics that of a mature mRNA molecule and, in the first case, consists of an open reading frame (ORF) that encodes the peptide of interest, two untranslated regions (UTRs) (one on each side of the coding sequence), a five-prime cap (5′ cap) and a poly-A tail [7]. saRNA molecules, on the other hand, include additional regions, specifically a 5′ and a 3′ conserved sequence element (CSE), non-structural proteins 1–4 (nsP1–4), usually derived from an alphavirus, and a subgenomic promoter. The nsP1–4 encodes an RNA-dependent RNA polymerase (RdRP) that recognizes the CSEs and amplifies the original RNA strand, causing an increase in the number of Ag copies produced into the cell [9,10].

Before reaching the cytoplasm, where it can be processed by the cellular translation machinery, resulting in the production of a protein that will afterward be subjected to the appropriate post-translational modifications, the mRNA molecule has to cross the lipid membrane barrier. In order to achieve that, two basic methods have been developed. The first one is ex vivo transfection of DCs with mRNA (frequently using electroporation) followed by their re-infusion in the bloodstream, while the second one is the parenteral injection of mRNA, naked or with a carrier (utilization of complexes with protamine, cationic nanoemulsion, modified dendrimer nanoparticles, protamine liposomes, polysaccharide particles and cationic lipid nanoparticles with or without cholesterol and polyethylene glycol -PEG-). Of course, the second procedure is less accurate regarding the determination of the cell type that will host the mRNA molecule [7].

Once the desired peptide epitope is produced into the APC, it can be demonstrated to T-cells through major histocompatibility complex (MHC) molecules (class I and II for CD8+ and CD4+ T-cells, respectively). mRNA vaccines have generally been proven able to induce stronger CD8+ T-cell responses than protein-based ones, as the presentation of endogenously produced Ags on MHC class I molecules seems to be more efficient [5,6,7]. Especially ex vivo transfected DCs are responsible for immune responses that are mainly cell-mediated and are consequently considered an ideal approach in cancer treatment. In addition to MHC class I-mediated presentation to CD4+ T-cells, direct exposure of B-cells to intact Ags by DCs also contributes to Ab production [7]. Moreover, in animal experiments, compared to DNA vaccines, lower immunization doses of mRNA are needed in order to cause an adequate neutralizing Ab (NAb) release [5,6,7]. 

## 3. Brief Presentation of Anti-SARS-CoV-2 mRNA Vaccines

### 3.1. BNT162b2

BNT162b2 is an mRNA vaccine against coronavirus disease 2019 (COVID-19), a condition caused by the severe acute respiratory syndrome coronavirus 2 (SARS-CoV-2). It was developed by Pfizer and BioNTech and selected among three others for phase 2/3 studies [8,9,10,11]. Finally, it was granted emergency use authorization in December 2020 by the World Health Organization (WHO) [11].

The SARS-CoV-2 mRNA has a length of 30 kb, and its OFRs are responsible for encoding at least four structural (spike -S-, membrane -M-, envelope -E- and nucleocapsid -N-) and 16 non-structural proteins [8]. The S protein binds to angiotensin-converting enzyme 2 (ACE2) receptors (ACE2-Rs) and mediates the mRNA integration into the targeted APCs, thus consisting of a promising vaccine candidate target. BNT162b2 is a nucleoside-modified mRNA molecule incorporated in lipid nanoparticles and encodes the full-length SARS-CoV-2 S protein with two proline mutations that ensure the preservation of the produced peptide in perfusion conformation. Exposure of the desired antigenic product on the cell surface can induce strong CD4+ and CD8+-mediated immune responses that protect from severe future SARS-CoV-2 infections [8,9,10,11]. 

The vaccine is given intramuscularly (IM) in two doses, 21 days apart [11,12]. A third booster dose is scheduled approximately 6 months after the two-dose regimen, while the administration of a fourth one (four months later) has been proven effective in reducing short-term COVID-19-related outcomes when tested in certain “at-risk” patient groups in several countries [13,14]. 

### 3.2. mRNA-1273 Vaccine

It is another anti-SARS-CoV-2 mRNA vaccine platform developed by Moderna with the contribution of the Vaccine Research Center at the National Institute of Allergy and Infectious Diseases (NIAID) and the Biomedical Advanced Research and Development Authority (BARDA) [15]. Its utilization in the United States was also initiated under a Food and Drug Association (FDA) emergency use authorization in April 2021 [16,17]. 

The vaccine mRNA molecule comprises the S1–S2 cleavage region (sequence enabling furin cleavage) and the transmembrane domain (region anchored to the viral membrane) of the SARS-CoV-2 S glycoprotein. It is stabilized on its perfusion conformation thanks to two proline substitutions at amino-acid positions 986 and 987, and lipid nanoparticles serve as a vehicle for its delivery into cells [18,19,20]. The vaccine is capable of inducing a robust anti-RBD and NAb response, as well as a considerable CD8+-mediated immunity activation [16]. According to the current guidelines, the first two mRNA-1273 doses are administered with a 28-day interval between them, while the first booster dose can be given at least three months after the completion of the first vaccination series [21]. 

## 4. Synopsis of Alternative Vaccine Platforms Used against COVID-19

### 4.1. Viral Vector-Based Vaccines

In viral vector vaccine manufacture, non-toxic (non-pathogenic or attenuated, either replicating or non-replicating) viruses are used as vectors. Adenoviruses, which are the vectors of preference in COVID-19 vaccine development, are generally a common choice, as they combine strong immunogenicity with a satisfactory safety profile. A complementary DNA (cDNA) sequence encoding a SARS-CoV-2-derived protein fragment is embedded in the vector after some parts of its genome have been removed. Once IM is injected, the inserted sequence replicates, resulting in the expression of the pathogenic peptide and the subsequent induction of cellular and humoral immunity activation [20,21,22].

ChAdOx1 nCoV-19 and Ad26.COV2.S, produced by Oxford/AstraZeneca and the Janssen Pharmaceutical Companies, respectively, are the only two representatives of this particular vaccine type [20,21,22,23]. Both of the previously mentioned platforms are based upon the integration of a SARS-CoV-2 protein S-encoding gene in the DNA of a selected vector (a modified, replication-deficient chimpanzee adenovirus in the first case and a recombinant, human-based, replication-deficient adenovirus serotype 26 in the second one) [16,17,18,19,20].

### 4.2. Inactivated Virus Vaccines

In order to produce inactivated virus vaccines, SARS-CoV-2 is initially cultivated into a susceptible cell line (such as Vero E6) in biosafety level 3 facilities [16,23,24]. Afterward, it is inactivated chemically (e.g., with formaldehyde, glutaraldehyde, β-propiolactone, ethylenimine, phenol, ascorbic acid, β-aminophenylketone, or diethylpyrocarbonate) or with the utilization of ultraviolet or gamma rays [20,21,22,23,24]. The vaccines are often adjuvanted and generally have the potential to induce broad (though sometimes marginal [20]) immune responses, both humoral and cellular, as they mediate exposure to a variety of viral antigens (e.g., proteins S, M, and N) [23]. The disadvantages of these platforms are mainly associated with their inability to produce robust memory responses [20], as well as with the possibility of inadequate viral inactivation, which could pose a danger to facility workers [23]. 

Examples in this category include the vaccines developed by Sinopharm (BBIBP-CorV, prepared after the isolation of an HB02 SARS-CoV-2 strain), Sinovac (CoronaVac, production based upon a CN2 strain, with the spike protein RBD being the dominant immunogen) [25] and Bharat Biotech (Covaxin, utilization of an NIV-2020-770 stain [24], formulation of the inactivated virus with a toll-like receptor 7/8 agonist molecule [20]).

### 4.3. Recombinant Protein-Based Vaccines

The development of NVX-CoV2373, the main representative of this category, is based upon the manufacture of a modified SARS-CoV-2 glycoprotein S-encoding gene, which is fused with a baculovirus. The recombinant viral molecules are used to infect selective moth cells, resulting in the production of full-length S proteins resembling those of SARS-CoV-2, which are then purified and inserted into nanoparticles. Adjuvants are often used. Administration of the vaccine in non-human mammals led to strong CD4+-driven B- and T-cell responses, as well as to the production of considerable levels of ACE2-R blocking Abs and SARS-CoV-2 Nabs [20,21,22].

## 5. CKD-Associated Immunological Aging

According to Kidney Disease Quality Outcome Initiative (K/DQOI), chronic kidney disease (CKD) is defined as kidney damage or glomerular filtration rate (GFR) < 60 mL/min/1.73 m^2^, which, regardless of the underlying cause, persists for ≥3 months [26]. CKD is a condition responsible for the acceleration of the normal aging process, with several mechanisms being involved. More specifically, uremia has been associated with defects in the balance between pro-oxidant and anti-oxidant factors, thus inducing an oxidative stress state. The production of reactive oxygen species (ROS) contributes to inflammation, which is maintained by moderate levels of proinflammatory mediators, such as C-reactive protein (CRP), interleukin-1 (IL-1), IL-6, and tumor necrosis factor α (TNF-α), that in turn aggravate CKD severity. Generally, the immune system is one of the most affected by chronic oxidative stress, with the induced immunosenescence contributing to the disruption of the body’s homeostasis. More recently, additional mechanisms implicated in the acceleration of the aging process have been described, with epigenetic factors and genomic damage being included [27].

### 5.1. Alterations in T-Lymphocytes

Regarding the immune system alterations observed in CKD, innate and adaptive immunity are both affected. Concerning the first one, there is an increase in the macrophage scavenger receptors, as well as in the mannose-binding lectin (MBL) sera levels. Monocytes and monocyte-derived DCs exhibit impaired endocytosis and maturation, leading to decreased Ag presentation and production of Ag-specific T-cells. Although white blood cell (WBC) numbers are normal, they have a poorer capacity for phagocytosis and a greater rate of apoptosis, while their leukocyte-integrin-mediated infiltration of inflamed tissues is also defective. As for adaptive immunity, there is a prevalence of T helper 1 (Th1) responses, with end-stage kidney disease (ESKD) patients having a reduced CD4+/CD8+ T-cell ratio (to a degree because the numbers of naïve and memory CD4+ T-cells drop, while those of CD8+ memory T-cells rise). Moreover, there is an increase in the number of CD4+CD28- highly differentiated T-cells and a decrease in regulatory T-cell (T-reg), naïve and central memory T-cell concentrations [28,29,30]. Alterations in the thymic function (possibly related to inflammation-induced lymphoid organ volume loss), which is normally responsible for the generation of the TCR repertoire and the control of autoreactive T-cells, are partially accountable for the changes observed in T-cell immunity. Indeed, the uremia-associated contraction of the circulating naïve T-cell compartment seems to result from a combination of a decrease in the recent thymus emigrant (RTE) cell numbers (defined by some authors as CD31+ naïve T-cells) and a reduction in the compensatory homeostatic proliferation rate [31,32,33]. Changes in T lymphocyte subpopulations in ESKD patients, compared to controls, are described in Table 1.

### 5.2. Alterations in B-lymphocytes

B-lymphocytes are affected to a greater degree than T-cells in ESKD, displaying a rather senescent phenotype with a gradual reduction in the expression of immunoglobin D (IgD) and CD27 molecules in memory populations. Progressively developing lymphopenia, which influences the total number of B-cells, is more prominent in the memory IgM-producing (IgD+ CD27+) subgroup [32]. Moreover, some authors have underlined the presence of a negative correlation between B1 (IgM- and IgA-secreting CD19+ CD5+ cells, important to innate immunity procedures) and B2 (CD19+ CD5- cells that produce IgG and finally differentiate into PCs) cell counts and markers of CKD progression (such as serum creatinine -SCr- and blood urea nitrogen -BUN- values) in elderly individuals. Reduced concentrations of B1 and B2 have also been linked to higher rates of all-cause mortality. The decrease in B-lymphocyte numbers can partially be attributed to their resistance to IL-7 and B-cell activating factor (BAFF), cytokines substantial to their differentiation and survival [34], which is in accordance with the observed low B-cell lymphoma 2 gene (bcl-2) expression levels in these individuals [35]. A factor majorly contributing to the previously described alterations in patients with impaired renal function is the accumulation of microbiota-derived metabolites, such as indoxyl sulfate, that bind to the aryl hydrocarbon receptor (AhR), inducing the translocation of the produced complex into the nucleus, where it regulates gene transcription. Normally, AhR serves as a resistor in the activation-mediated B-cell modifications, as it suppresses class switching and inhibits differentiation of B-cells into Ab-secreting PCs [35,36]. As anticipated, B-cell Ab-producing capacity is diminished, thus rendering the humoral immunity severely impaired [31,32,34,35,37,38].

Due to all the parameters analyzed above, CKD patients, especially those in stages 4 and 5, are more susceptible to infections (that often present with severe manifestations), and they also exhibit reduced vaccination effectiveness. This is why various modifications to the general population guidelines regarding vaccination protocols have been proposed (mostly in order to re-adjust the vaccination doses and the relevant intervals), with some differentiations from country to country that mainly have to do with the local epidemiological data. Nevertheless, we could mention some universal practices: First, ESKD patients and RT recipients should not be vaccinated with live-attenuated vaccines. Second, in the case of programmed RT, the immunological status of the patient has to be checked, and all the necessary vaccinations need to be completed at least four weeks prior to the procedure. Lastly, immunization (evaluated by serological testing) against hepatitis B virus (HBV) is recommended in all adults with progressive CKD and GFR < 30 mL/min/1.73 m^2^ [37]. 

## 6. Characteristics of SARS-CoV-2 Infection and Anti-SARS-CoV-2 Vaccination in CKD Patients

### 6.1. Impact of CKD-Associated Immunological Changes to COVID-19 Manifestations

As anticipated, with a combination of immunological deficits and abnormalities being present, patients with CKD are more prone to severe COVID-19 manifestations. 

The presence of end-stage kidney disease, especially when patients are on dialysis, has been recognized as one of the most vicious factors, predisposing to worse outcomes. A wide range of disease severity and complications has been manifested, such as severe pneumonia, liver failure, gastrointestinal symptoms, such as diarrhea and gastrointestinal bleeding, cardiovascular events, including atrial fibrillation and QTc interval prolongation, and acute respiratory distress syndrome (ARDS). Age, comorbidity, for instance, ischemic heart disease, and severe symptomatology at diagnosis are factors associated with worse outcomes, ARDS, and increased mortality [39,40,41]. Immunological alterations in these patients also seem to play a substantial role in the outcome of infection. The repository of TCRs in naïve T-cell populations is reduced, and T-cell-mediated intracellular signaling is less effective, thus rendering their function impaired. Additionally, plasmacytoid DCs (pDCs), the major source of type I interferon (IFN) [42], a chemokine of main importance in the combat against COVID-19, are decreased. All the previous changes are responsible for delayed virus clearance and prolonged stimulation of SARS-CoV-2-reactive memory T-cells. As Tregs are also defective and the numbers of naïve T-cells are low, the ability of the immune system to control this large-scale expansion of highly reactive T-cells and the consequent cytokine storm is restricted, with the collateral damage caused being significant. Interestingly, in the case of COVID-19, the intensity of the immune response is possibly much worse at the tissue level compared to peripheral blood, as the expansion of reactive effector T-cells seems to be more extended in the lung parenchyma [33]. 

### 6.2. CKD-Associated Immune Changes and Response to Vaccination against COVID-19

Although the determination of anti-SARS-CoV-2 Ab titters is a quick way of assessing the patient’s response to immunization, cellular immunity assays are also necessary in order to form a more comprehensive image regarding their condition. Cellular immunity activation is of great importance in the defense against SARS-CoV-2 since it can significantly reduce the risk of infection, with cytotoxic CD8+ T-cells being a potential asset in terms of viral clearance [43]. According to the results of recent trials, both mRNA and viral vector vaccines have the potential to induce sufficiently strong humoral and cellular responses in the general population. More specifically, it has been proven that IM administration of two mRNA vaccine doses can mediate a robust S protein-specific Ab production and a satisfactory CD4+ and CD8+ T-cell activation, while the viral-vectored ChAdOx1 nCoV-19 can cause sufficient NAb development and T-cell mobilization in the great majority of vaccines [43,44]. On the contrary, protein subunit and inactivated viral vaccines are poor inducers of CD8+ T-cells and mainly promote CD4+ Th responses and Ab formation [45]. Interestingly, based on phase 3 trial results, Windpessl et al. stated that, since mRNA vaccines had prevented COVID-19 in a larger proportion of participants compared to ChAdOx nCoV-19 [15,45,46], they could possibly be a more suitable solution for immunocompromised individuals [43]. 

In their review, Carr et al. support that a significant proportion of dialysis patients display Ab development after two vaccine doses; nevertheless, humoral immunity activation among them is still deficient in comparison to that of the general population, rendering them more dependent on the administration of booster doses in order to maintain protective concentrations [47]. Indeed, according to the meta-analysis of Ma et al., dialysis patients had an 18% lesser possibility of producing anti-SARS-CoV-2 Abs, while no statistically significant difference was observed between peritoneal dialysis (PD) patients and healthy individuals [48]. Other investigators, however, described a similar humoral response, estimated by the anti-spike IgG levels, between PD and HD patients, with almost 50% of both groups developing detectable levels after the first dose and increasing to 90% following the second dose of vaccination [49].

It is, however, important to underline that, as their Ab-producing ability is markedly superior to that of renal transplant recipients (RTRs), completion of the vaccination scheme against SARS-CoV-2 prior to renal transplantation (RT) should be seriously considered in persons planning to receive a graft [47].

Cellular immunity activation has also been evaluated in various studies. In the research of Bertrand et al., almost all HD participants had developed a detectable spike-reactive T-cell response after the completion of the two-dose BNT162b2 series at levels comparable to those recorded in the general population [50]. The findings described above were consistent with those of Sattler et al., according to whom most individuals in HD have the potential to mount cellular and humoral anti-SARS-CoV-2 response after the administration of an mRNA vaccine [51]. However, Van Praet et al. stated that, in dialysis patients in their study, after vaccination with the mRNA-1273, both the mean IFN-γ release by circulating CD4+ and CD8+ T-cells induced by SARS-CoV-2 glycoprotein stimulation and the percentage of individuals having IFN-γ production above the cut-off value, was significantly lower than those of healthy controls. The same authors also observed that anti-SARS-CoV-2 response was even more compromised in the subgroup of hepatitis B virus (HBV) vaccination non-responders and supported that mRNA-1273 is more immunogenic than BNT162b2 in HD patients, possibly due to the presence of a higher mRNA dose in it [52]. Interestingly, a German team of researchers noticed that after two doses of BNT162b2, people in dialysis tend to display increased levels of proinflammatory cytokines (such as chemokine C-C motif ligand 2 (CCL-2) and IL-8), which reflects a more profound innate immune response activation (with the participation of monocytes, neutrophils, and endothelial cells), accompanied by an impaired SARS-CoV-2-specific T-cell response [53].

Nevertheless, even though vaccination against SARS-CoV-2 in individuals with ESKD or in HD mediates weakened and defective immune responses compared to the general population, it still offers remarkable clinical results, as it reduces the infection, hospitalization, and death rates in that group of patients [54]. 

## 7. Can Successful Renal Transplantation Reinstate the Immune Profile?

Lymphocyte number, which is significantly eliminated in CKD patients, returns to normal levels soon after transplantation; however, there are functional and population diversities. In RTRs, the thymic output is unchanged and lower than normal, as indicated by the stable numbers of CD31+ naïve T-cells and T-cell receptor excision circles (TREC) concentrations [55]. The latter are small circular DNA episomes formed during the variability, diversity, and joining -V(D)J- recombination of the TCR genes in the thymus and can be used to assess thymic function [55,56]. At the same time, the relative telomere length (RTL) of naïve T-cells is shortened, while that of CD4+ and CD8+ memory T-cells remains unaltered. These findings are consistent with the homeostatic proliferation of naïve T-cells in response to cytokines such as IL-7 and to stimuli produced by self-Ag-displaying APCs. That procedure is possibly compensatory to a transient decrease in memory T-cell numbers, which is perhaps caused by immunosuppressive regimens and can be restored within several months. The fact that RT is unable to reinstate a healthy T-cell profile seems to result mainly from the accumulation of irreversible uremia-induced epigenetic changes in ESKD patients, while the possibility that immunosuppressive treatment (IST) has the potential to impede the repair of the thymus cannot be rejected [55]. In their recent research, Vagiotas et al. described that RT may have different influences on different T lymphocyte subtypes. According to their findings, while regulatory T-cells (Tregs) show an early response by increasing their number in the first three months of follow-up, and natural killer (NK) cells, which are increased in hemodialysis patients, return to normal levels, the situation with CD28null cells is not the same. This type of T-lymphocytes does not seem to benefit from RT, and regardless of renal function restoration, their peripheral population remains high [57]. Other studies have described a temporal reduction in CD4+, CD8+, and NK cells during the first two weeks following RT, which were restored to normal levels thereafter [58].

Alterations in lymphocyte subpopulations in RTRs compared to those observed in ESKD patients are demonstrated in Table 1.

Very importantly, the presence of Panel Reactive Antibodies (PRA) was found to have a central role in the restoration of patients’ immune profiles after RT [57]. High PRA levels are usually the consequence of previous exposure to foreign human leucocyte antigens (HLA) due to pregnancy, blood transfusion, or previous RT, and they represent increased quantities of preformed donor-specific antibodies (DSA) [59,60,61]. Patients carrying HLA-DSAs usually have increased blood levels of proinflammatory cytokines, which act by obstructing the reinstatement of lymphocyte function after successful RT. Although it is not yet clear whether the production of such cytokines is due to direct endothelial cell activation by DSAs or ensues as a consequence of the interaction between endothelial and circulating cells, including NK cells and macrophages, many investigators have shown that the presence of HLA-DSAs can lead to immune activation. Thus, positive HLA-DSAs can predict an allo-immune activation, which is though not always accompanied by pathological signs of rejection [62]. 

The direct effects of lymphocyte disturbances, such as the development of infections, graft rejection, malignancies, and acceleration of cardiovascular disease, are usually obvious and predictable. Some recent studies suggest relying on INF-γ-expressing T-lymphocytes in order to regulate IST [58], while others have indicated that the presence of lymphopenia is a predive factor for polyomavirus hominis 1 (BK) viremia [63].

There are, however, situations not widely recognizable (e.g., enhanced senescence of the graft followed by tubular atrophy and interstitial fibrosis) that lead to chronic allograft dysfunction. Experimental studies have shown that inhibition of inflammatory infiltration by macrophages and CD8+ lymphocytes and reduction in proinflammatory cytokines, such as monocyte chemoattractant protein-1 (MCP-1), IL-1b, and TNF-a, with the use of mammalian/mechanistic target of rapamycin (mTOR) inhibitors, may prevent the emergence of phenotypic changes linked to senescence and chronic graft dysfunction [64]. 

## 8. COVID-19 Infection in Renal Transplant Recipients

With their ability to produce an efficient immune response being significantly compromised, it is not a surprise that RTRs have a higher incidence of SARS-CoV-2 infection compared to the general population, as well as an increased risk of adverse COVID-19 outcomes [65,66,67,68,69]. A worse disease course is definitely anticipated during the early post-transplantation period and among recipients of deceased donor transplants [68]. Many researchers have pointed out that the prognosis in hospitalized RTRs is poorer, the clinical progression faster, and the mortality rates significantly higher, while in a considerable proportion of them, the disease is complicated with acute kidney injury (AKI). Potential mechanisms leading to the latter vary from direct parenchymal damage and inflammation-induced microangiopathy to acute rejection (due to the reduction in immunosuppressants to subtherapeutic levels) and calcineurin inhibitor (CNI) toxicity (resulting from their interaction with other drugs) [65,66,67,68,69]. The clinical image of the disease may differ from that in healthy individuals, with notably smaller percentages of RTRs being reported with fever and a greater proportion of them experiencing malaise and respiratory and gastrointestinal symptoms [70]. Lower numbers of CD3+, CD4+, and CD8+ T-cells have also been observed among these patients [65]. Additionally, non-white ethnicity and the coexistence of conditions such as chronic obstructive pulmonary disease (COPD), asthma, diabetes mellitus (DM), and obesity have been linked to greater chances of developing COVID-19 [66]. Several approaches have been attempted, including discontinuation of antiproliferative agents such as mycophenolate mofetil (MMF) (universally applied even in mild disease) and administration of antivirals, corticosteroids, hydroxychloroquine, and tocilizumab. Finally, the WHO supported that the systemic use of corticosteroids in severe and life-threatening diseases has turned out to be beneficial in many cases and is consequently advised [68]. 

## 9. Modifications of the Vaccination Schedules in Renal Transplant Recipients

Because of their impaired immune system status, which is, of course, additionally burdened due to the chronic use of immunosuppressives, especially steroids and mycophenolate mofetil [71], RTRs have a poorer response to vaccination with lower rates of seroconversion, reduced Ab titters and shorter periods of protective immunity compared to healthy individuals.

Several prospective studies have confirmed a significantly lower seroconversion rate in RTR following anti-SARS-CoV-2 vaccination, estimated at 50%, compared to healthy individuals, almost 100%. Factors that may be responsible for the poor response are elder age, short transplantation vintage, immunosuppression, and type of mRNA vaccination [71]. 

Immunogenic parameters, apparently correlated to immunosuppression and renal function, are probably implicated, and this becomes possible as the administration of the fourth and recently fifth dose of vaccination increases the response rate among RT patients. 

However, in RTR, who persistently do not respond even after booster vaccination dosing, modulation of immunosuppression may be considered as well as passive immunoprophylaxis [71,72,73].

In not previously immunized patients, the administration of inactivated vaccines can be initiated at least six months after the transplantation, while the live-attenuated ones are generally contraindicated. Unfortunately, the guidelines regarding booster doses are deficient [74]. Some of the vaccination guidelines, as have been proposed previously [75,76,77,78,79] are presented in Table 2. 

## 10. Response of Renal Transplant Patients to COVID-19 Vaccination

When it comes to vaccination against COVID-19, the condition is not different. After two doses, seroconversion percentages, as well as Ab concentrations, are significantly lower among RTRs compared to healthy individuals and even ESKD patients on dialysis [48]. In the same context, spike-specific T-cell immunity is detected in a notably lower proportion of RTRs compared to the percentages recorded even in HD patients [50,51], with the numbers of short-lived effector Ths being increased and the concentrations of memory cells displaying a notable reduction [51]. Regarding the interpretation of the poor vaccine-specific Th activation, Sattler et al. have supported that it possibly has to do with the downregulation of pathways implicated in cellular activation, cytokine signaling, and metabolism, which results from the utilization of immunomodulatory regimens that disrupt several signaling mechanisms (e.g., the IL-2/signal transducer and activator of transcription (STAT) pathway). Additionally, spike-specific CD8+ T-cells are detectable in even fewer vaccinated RTRs (possibly due to the diminished IL-2 production by spike-specific CD4+ cells and the downregulation of other cytokine signaling pathways) [51]. Nevertheless, the presence of specific T-cell response in Ab-negative individuals can still provide some protection against SARS-CoV-2, as it limits the extent of viral replication [50]. 

As mentioned above, this poor immunogenicity status has mainly been attributed to the use of IST after RT, with all of the regimens of the typical triad most patients receive being potentially responsible. More specifically, MMF (considered majorly culpable for the insufficient Ab development) impedes B-cell proliferation and maturation processes and intervenes in Th cell functions (e.g., halts the proliferation of follicular Th cells), CNIs inhibit T-cell activation (by affecting several metabolic pathways, including glycolysis, as well as the function of nuclear factor kappa beta (NFκB) [51]) and corticosteroids disrupt monocyte and macrophage activities [48]. In that context, medication alterations, such as dosage reduction or temporary discontinuation of MMF, have been proposed by some researchers in order to improve vaccine efficacy. However, such practices could increase the risk of graft rejection by triggering DSA generation. It should be noted here that lower anti-SARS-CoV-2 neutralizing Ab titers after the completion of two vaccination doses have been linked to older age, presence of DM, higher levels of immunosuppression, reduced eGFR values, and inability to produce detectable concentrations of Abs after the administration of the first dose. On the contrary, the previous history of COVID-19 seems to be a principally contributing factor to successful seroconversion, and since approaches such as the utilization of adjuvants have not yet been tested in mRNA vaccines, booster doses seem like a reasonable way to increase immunogenicity in that group of patients [80]. 

As with CKD patients [54], even though the response of RTRs to vaccination is suboptimal, it definitely improves their clinical outcomes. It has been observed that among transplant patients with similar risk factors, vaccinated ones tend to display lower rates of hospital entries and deaths, as well as shorter hospitalization periods due to COVID-19 [81].

## 11. Proposed Strategies to Improve COVID-19 Outcome in CKD and RTR

Main strategies to improve the outcome of a possible COVID-19 infection in CKD or RTR include [82,83]:

- Precautionary actions to reduce the risk of exposure to SARS-CoV-2;
- Close monitoring of the CKD or RT patients suspected to have COVID-19;
- Nutritional support;
- Modification of immunosuppression;
- Booster vaccination doses;
- Prophylactic use of mAbs in patients with poor response to vaccination.

## 12. Conclusions

Given the susceptibility of CKD patients’ RTRs toward more severe COVID-19 disease manifestations, combined with their suboptimal vaccination-associated immunization performance, it is clear that there is a great need for research efforts to focus on the response of that particularly vulnerable population group to the administration of the novel vaccines against the SARS-CoV-2 virus.

## Figures and Tables

**Table 1:** Summary of alterations in lymphocyte subpopulations of ESKD patients and RTRs, compared to healthy controls.

	ESKD	Renal Transplantation	
		Early Post RT *	Late Post RT
**CD4+ cells**		↑ **	
**Naive lymphocytes**			
CD4+CD31+		↑	
CD4+CD45RA+CCR7+		↑	
**Memory lymphocytes**			
CD4+CD45RA-CCR7+ (CM)			
CD4+CD45RA-CCR7- (EM)		↑	↑
**Aged lymphocytes**			
CD4+CD45RA+CCR7-			
CD4+CD28-	↑	↑	↑↑
**Exhausted lymphocytes**			
CD4+PD1+			
**CD8+ cells**			
**Naive lymphocytes**			
CD8+CD31+		↑	
CD8+CD45RA+CCR7+		↑	
**Memory lymphocytes**			
CD8+CD45RA-CCR7+ (CM)			
CD8+CD45RA-CCR7- (EM)			
**Aged lymphocytes**			
CD8+CD45RA+CCR7-			↑
CD8+CD28-		↑	↑
**Exhausted lymphocytes**			
CD8+PD1+		↑	↑
**Natural killer cells**	↑		
**Regulatory T-lymphocytes**		↑	

Abbreviations: CCR: C-C chemokine receptor, CD: cluster of differentiation, CM: central memory, EM: effector memory, ESKD: end-stage kidney disease, PD: programmed death, RT: renal transplantation. * We used the terms early or late post RT to describe time periods of <3 months or >3 months following renal transplant. ** All arrows show changes compared to healthy individuals.

**Table 2:** Indicative vaccination guidelines for adult CKD pts and RTRs.

Vaccine	CKD	RTRs
HBV recombinant	CKD stages 3–4: • Recombivax 10 μg/mL: 3 doses (mo: 0, 1, 6) • Engerix-B 20 μg/mL: 4 doses (mo: 0, 1, 2, 6) HD and other immunocompromised pts ≥ 20 y/o: • Recombivax 40 μg/mL: 3 doses (mo: 0, 1, 6) • Engerix-B 20 μg/mL: 4 doses (DOUBLE amount in each, mo: 0, 1, 2, 6) Serologic testing 1–2 mo after last dose of vaccine series -> if anti-HBs < 10 mIU/mL: perform a 2nd vaccination series → if again anti-HBs < 10 mIU/mL → test for HBsAg • If HBsAg (+): HBV treatment and management • If HBsAg (−): susceptible to HBV—HBIG postexposure profylaxis In HD pts test annually for anti-HBs: if anti-HBs < 10 mIU/mL → administer booster dose	• Vaccinate if anti-HBs < 10 mIU/mL—preferably > 3 mo post KTx (↓ IST dosage) • Test every 6–12 mo for anti-HBs: if anti-HBs < 10 mIU/mL or expected to be < 10 mIU/mL within the next 3–6 mo → administer booster dose
TIV (inactivated)	Routine annual vaccination—ideally before influenza season onset	• Routine annual vaccination • Use of adjuvated vaccines not advised (causes ↑ anti-HLA Abs)
Pneumococcal vaccines	• CKD pts considered at high risk for IPD—combination of PCV13 and PPSV23 administration advised • Pts naïve to pneumococcal immunization: 1 dose of PVC13, 1 dose of PPSV23 ≥ 8 wks later, 2nd dose (booster) of PPSV23 ≥ 5 yrs after the 1st PPSV23 dose • Variations in guidelines for previously immunized individuals	• Both PCV13 and PPSV23 considered safe • Same schedule as for general adults • Pts naïve to pneumococcal immunization: 1st immunization with PCV13 followed by PPSV23 ≥ 8 wks later
HAV inactivated	• Not universally recommended- consider in CKD pts who travel in endemic areas, with CLD, HCV and/or HIV infection history, IV drug users, etc. • 2 doses (mo: 0, 6–12)	Can be administered to high risk pts
TdaP and Td	Pts naïve to immunization: 3 doses (mo: 0, 1, 6–12) including 1 dose of TdaP—booster with Td every 10 yrs	Pts naïve to immunization: 3 doses (mo: 0, 1, 6–12) including 1 dose of TdaP—booster with Td every 10 yrs
VAR	Pts naïve to immunization: 2 doses with an interval of 4–8 wks	Contraindicated
RZV	• Not universally recommended for CKD pts • Pts > 50 yrs (regardless of past herpes zoster infection or ZVL administration): 2 doses with an interval of 2–6 mo—more efficient than ZVL	RZV possibly safe—ZVL contraindicated

Abbreviations: antibodies, anti-HBs: hepatitis B surface antibody, anti-HLA: anti-human leukocyte antigen, CKD: chronic kidney disease, CLD: chronic liver disease, HBIG: hepatitis B immune globulin, HBsAg: hepatitis B surface antigen, HAV: hepatitis A virus, HBV: hepatitis B virus, HCV: hepatitis C virus, HD: hemodialysis, HIV: human immunodeficiency virus, IPD: invasive pneumococcal disease, IST: immunosuppressive treatment, IV: intravenous, KTx: kidney transplantation, PCV13: pneumococcal conjugate vaccine, PPSV23: pneumococcal polysaccharide vaccine, pts: patients, RTRs: renal transplant recipients, RZV: recombinant zoster vaccine, Td: tetanus-diphtheria vaccine, TdaP: tetanus-diphtheria-acellular pertussis vaccine, TIV: trivalent influenza vaccine, VAR: varicella, wks: weeks, yrs: years, y/o: years old, ZVL: herpes zoster live-attenuated vaccine. All arrows in the text signify the suggestion deriving from the described event.

## References

[1] Gansevoort R.T., Hilbrands L.B. (2020). CKD is a key risk factor for COVID-19 mortality. Nat. Rev. Nephrol..

[2] Puchalska-Reglińska E., Dębska-Ślizień A., Biedunkiewicz B., Tylicki P., Polewska K., Jagodziński P., Rutkowski B., Gellert R., Tylicki L. (2021). Extremely high mortality in COVID-19 hemodialyzed patients in before anty-SARS-CoV-2 vaccination era. The first large database from Poland. Pol. Arch. Intern. Med..

[3] Council E.-E., Ortiz A., Cozzolino M., Fliser D., Fouque D., Goumenos D., Massy Z.A., Rosenkranz A.R., Rychlık I., Soler M.J. (2021). Chronic kidney disease is a key risk factor for severe COVID-19: A call to action by the ERA-EDTA. Nephrol. Dial. Transplant..

[4] Pollard A.J., Bijker E.M. (2020). A guide to vaccinology: From basic principles to new developments. Nat. Rev. Immunol..

[5] Qin F., Xia F., Chen H., Cui B., Feng Y., Zhang P., Chen J., Luo M. (2021). A Guide to Nucleic Acid Vaccines in the Prevention and Treatment of Infectious Diseases and Cancers: From Basic Principles to Current Applications. Front. Cell Dev. Biol..

[6] Rapaka R., Cross A., McArthur M. (2021). Using Adjuvants to Drive T Cell Responses for Next-Generation Infectious Disease Vaccines. Vaccines.

[7] Pardi N., Hogan M., Porter F., Weissman D. (2018). mRNA vaccines—A new era in vaccinology. Nat. Rev. Drug Discov..

[8] Teo S.P. (2021). Review of COVID-19 mRNA Vaccines: BNT162b2 and mRNA-1273. J. Pharm. Pract..

[9] Bloom K., van den Berg F., Arbuthnot P. (2020). Self-amplifying RNA vaccines for infectious diseases. Gene Ther..

[10] Blakney A. (2021). The next generation of RNA vaccines: Self-amplifying RNA. Biochemist.

[11] Lamb Y.N. (2021). BNT162b2 mRNA COVID-19 Vaccine: First Approval. Drugs.

[12] Savina K., Sreekumar R., Soonu V.K., Variyar E.J. (2022). Various vaccine platforms in the field of COVID-19. Beni-Suef Univ. J. Basic Appl. Sci..

[13] Gazit S., Saciuk Y., Perez G., Peretz A., Pitzer V., Patalon T. (2022). Relative Effectiveness of Four Doses Compared to Three Dose of the BNT162b2 Vaccine in Israel. medRxiv.

[14] Magen O., Waxman J.G., Makov-Assif M., Vered R., Dicker D., Hernán M.A., Lipsitch M., Reis B.Y., Balicer R.D., Dagan N. (2022). Fourth Dose of BNT162b2 mRNA COVID-19 Vaccine in a Nationwide Setting. N. Engl. J. Med..

[15] Baden L., El Sahly H., Essink B., Kotloff K., Frey S., Novak R., Diemert D., Spector S.A., Rouphael N., Creech C.B. (2021). Efficacy and Safety of the mRNA-1273 SARS-CoV-2 Vaccine. N. Engl. J. Med..

[16] Francis A.I., Ghany S., Gilkes T., Umakanthan S. (2021). Review of COVID-19 vaccine subtypes, efficacy and geographical distributions. Postgrad. Med. J..

[17] Pawlowski C., Lenehan P., Puranik A., Agarwal V., Venkatakrishnan A., Niesen M.J., O’Horo J.C., Virk A., Swift M.D., Badley A.D. (2021). FDA-authorized mRNA COVID-19 vaccines are effective per real-world evidence synthesized across a multi-state health system. Med.

[18] Corbett K.S., Edwards D.K., Leist S.R., Abiona O.M., Boyoglu-Barnum S., Gillespie R.A., Himansu S., Schäfer A., Ziwawo C.T., DiPiazza A.T. (2020). SARS-CoV-2 mRNA vaccine design enabled by prototype pathogen preparedness. Nature.

[19] Huang Y., Yang C., Xu X., Xu W., Liu S. (2020). Structural and functional properties of SARS-CoV-2 spike protein: Potential antivirus drug development for COVID-19. Acta Pharmacol. Sinica.

[20] Khandker S.S., Godman B., Jawad I., Meghla B.A., Tisha T.A., Khondoker M.U., Haq A., Charan J., Talukder A.A., Azmuda N. (2021). A Systematic Review on COVID-19 Vaccine Strategies, Their Effectiveness, and Issues. Vaccines.

[21] Luxi N., Giovanazzi A., Capuano A., Crisafulli S., Cutroneo P.M., Fantini M.P., Ferrajolo C., Moretti U., Poluzzi E., Raschi E. (2021). COVID-19 Vaccination in Pregnancy, Paediatrics, Immunocompromised Patients, and Persons with History of Allergy or Prior SARS-CoV-2 Infection: Overview of Current Recommendations and Pre- and Post-Marketing Evidence for Vaccine Efficacy and Safety. Drug Saf..

[22] Zhang Z., Shen Q., Chang H. (2022). Vaccines for COVID-19: A Systematic Review of Immunogenicity, Current Development, and Future Prospects. Front. Immunol..

[23] Nagy A., Alhatlani B. (2021). An overview of current COVID-19 vaccine platforms. Comput. Struct. Biotechnol. J..

[24] Khoshnood S., Arshadi M., Akrami S., Koupaei M., Ghahramanpour H., Shariati A., Sadeghifard N., Heidary M. (2022). An overview on inactivated and live-attenuated SARS-CoV-2 vaccines. J. Clin. Lab. Anal..

[25] Hotez P.J., Bottazzi M.E. (2022). Whole Inactivated Virus and Protein-Based COVID-19 Vaccines. Annu. Rev. Med..

[26] Levey A.S., Eckardt K.-U., Tsukamoto Y., Levin A., Coresh J., Rossert J., Zeeuw D.D., Hostetter T.H., Lameire N., Eknoyan G. (2005). Definition and classification of chronic kidney disease: A position statement from Kidney Disease: Improving Global Outcomes (KDIGO). Kidney Int..

[27] Figuer A., Bodega G., Tato P., Valera G., Serroukh N., Ceprian N., de Sequera P., Morales E., Carracedo J., Ramírez R. (2021). Premature Aging in Chronic Kidney Disease: The Outcome of Persistent Inflammation beyond the Bounds. Int. J. Environ. Res. Public Health.

[28] Sampani E., Vagiotas L., Daikidou D., Nikolaidou V., Xochelli A., Kasimatis E., Lioulios G., Dimitriadis C., Fylaktou A., Papagianni A. (2022). End stage renal disease has an early and continuous detrimental effect on regulatory T cells. Nephrology.

[29] Sampani E., Daikidou D.-V., Lioulios G., Xochelli A., Mitsoglou Z., Nikolaidou V., Dimitriadis C., Fylaktou A., Papagianni A., Stangou M. (2021). CD28null and Regulatory T Cells Are Substantially Disrupted in Patients with End-Stage Renal Disease Due to Diabetes Mellitus. Int. J. Mol. Sci..

[30] Sampani E., Stangou M., Daikidou D., Nikolaidou V., Asouchidou D., Dimitriadis C., Lioulios G., Xochelli A., Fylaktou A., Papagianni A. (2021). Influence of end stage renal disease on CD28 expression and T-cell immunity. Nephrology.

[31] Lioulios G., Fylaktou A., Papagianni A., Stangou M. (2021). T cell markers recount the course of immunosenescence in healthy individuals and chronic kidney disease. Clin. Immunol..

[32] Lioulios G., Fylaktou A., Xochelli A., Sampani E., Tsouchnikas I., Giamalis P., Daikidou D.-V., Nikolaidou V., Papagianni A., Theodorou I. (2022). Clustering of End Stage Renal Disease Patients by Dimensionality Reduction Algorithms According to Lymphocyte Senescence Markers. Front. Immunol..

[33] Betjes M.G.H. (2021). Uremia-Associated Immunological Aging and Severity of COVID-19 Infection. Front. Med..

[34] Lin J., Tang W., Liu W., Yu F., Wu Y., Fang X., Zhou M., Hao W., Hu W. (2020). Decreased B1 and B2 Lymphocytes Are Associated With Mortality in Elderly Patients With Chronic Kidney Diseases. Front. Med..

[35] Espi M., Koppe L., Fouque D., Thaunat O. (2020). Chronic Kidney Disease-Associated Immune Dysfunctions: Impact of Protein-Bound Uremic Retention Solutes on Immune Cells. Toxins.

[36] Vaidyanathan B., Chaudhry A., Yewdell W.T., Angeletti D., Yen W.-F., Wheatley A., Bradfield C.A., McDermott A.B., Yewdell J.W., Rudensky A.Y. (2016). The aryl hydrocarbon receptor controls cell-fate decisions in B cells. J. Exp. Med..

[37] Haddiya I. (2020). Current Knowledge of Vaccinations in Chronic Kidney Disease Patients. Int. J. Nephrol. Renov. Dis..

[38] Schroth J., Thiemermann C., Henson S.M. (2020). Senescence and the Aging Immune System as Major Drivers of Chronic Kidney Disease. Front. Cell Dev. Biol..

[39] Alberici F., Delbarba E., Manenti C., Econimo L., Valerio F., Pola A., Maffei C., Possenti S., Lucca B., Cortinovis R. (2020). A report from the Brescia Renal COVID Task Force on the clinical characteristics and short-term outcome of hemodialysis patients with SARS-CoV-2 infection. Kidney Int..

[40] Tylicki P., Polewska K., Och A., Susmarska A., Puchalska-Reglińska E., Parczewska A., Biedunkiewicz B., Szabat K., Renke M., Tylicki L. (2022). Angiotensin Converting Enzyme Inhibitors May Increase While Active Vitamin D May Decrease the Risk of Severe Pneumonia in SARS-CoV-2 Infected Patients with Chronic Kidney Disease on Maintenance Hemodialysis. Viruses.

[41] Yang X., Yu Y., Xu J., Shu H., Xia J., Liu H., Wu Y., Zhang L., Yu Z., Fang M. (2020). Clinical course and outcomes of critically ill patients with SARS-CoV-2 pneumonia in Wuhan, China: A single-centered, retrospective, observational study. Lancet Respir. Med..

[42] Fitzgerald-Bocarsly P., Dai J., Singh S. (2008). Plasmacytoid dendritic cells and type I IFN: 50 years of convergent history. Cytokine Growth Factor Rev..

[43] Windpessl M., Bruchfeld A., Anders H., Kramer H., Waldman M., Renia L., Ng L.F., Xing Z., Kronbichler A. (2021). COVID-19 vaccines and kidney disease. Nat. Rev. Nephrol..

[44] Jeyanathan M., Afkhami S., Smaill F., Miller M.S., Lichty B.D., Xing Z. (2020). Immunological considerations for COVID-19 vaccine strategies. Nat. Rev. Immunol..

[45] Polack F.P., Thomas S.J., Kitchin N., Absalon J., Gurtman A., Lockhart S., Perez J.L., Pérez Marc G., Moreira E.D., Zerbini C. (2020). Safety and efficacy of the BNT162b2 mRNA COVID-19 vaccine. N. Engl. J. Med..

[46] Voysey M., Clemens S., Madhi S., Weckx L., Folegatti P., Aley P., Angus B., Baillie V.L., Barnabas S.L., Bhorat Q.E. (2021). Safety and efficacy of the ChAdOx1 nCoV-19 vaccine (AZD1222) against SARS-CoV-2: An interim analysis of four randomised controlled trials in Brazil, South Africa, and the UK. Lancet.

[47] Carr E., Kronbichler A., Graham-Brown M., Abra G., Argyropoulos C., Harper L., Lerma E.V., Suri R.S., Topf J., Willicombe M. (2021). Review of Early Immune Response to SARS-CoV-2 Vaccination Among Patients With CKD. Kidney Int. Rep..

[48] Ma B.M., Tam A.R., Chan K.W., Ma M.K.M., Hung I.F.N., Yap D.Y.H., Chan T.M. (2022). Immunogenicity and Safety of COVID-19 Vaccines in Patients Receiving Renal Replacement Therapy: A Systematic Review and Meta-Analysis. Front. Med..

[49] Patecki M., Merscher S., Dumann H., Bernhardt W., Dopfer-Jablonka A., Cossmann A., Stankov M.V., Einecke G., Haller H., Schlieper G. (2021). Similar humoral immune responses in peritoneal dialysis and haemodialysis patients after two doses of the SARS-CoV-2 vaccine BNT162b2. Perit. Dial. Int. J. Int. Soc. Perit. Dial..

[50] Bertrand D., Hamzaoui M., Lemée V., Lamulle J., Hanoy M., Laurent C., Lebourg L., Etienne I., Lemoine M., Le Roy F. (2021). Antibody and T Cell Response to SARS-CoV-2 Messenger RNA BNT162b2 Vaccine in Kidney Transplant Recipients and Hemodialysis Patients. J. Am. Soc. Nephrol..

[51] Sattler A., Schrezenmeier E., Weber U., Potekhin A., Bachmann F., Straub-Hohenbleicher H., Budde K., Storz E., Proß V., Bergmann Y. (2021). Impaired humoral and cellular immunity after SARS-CoV-2 BNT162b2 (tozinameran) prime-boost vaccination in kidney transplant recipients. J. Clin. Investig..

[52] Van Praet J., Reynders M., De Bacquer D., Viaene L., Schoutteten M., Caluwé R., Doubel P., Heylen L., De Bel A.V., Van Vlem B. (2021). Predictors and Dynamics of the Humoral and Cellular Immune Response to SARS-CoV-2 mRNA Vaccines in Hemodialysis Patients: A Multicenter Observational Study. J. Am. Soc. Nephrol..

[53] Strengert M., Becker M., Ramos G., Dulovic A., Gruber J., Juengling J., Lürken K., Beigel A., Wrenger E., Lonnemann G. (2021). Cellular and humoral immunogenicity of a SARS-CoV-2 mRNA vaccine in patients on haemodialysis. EBioMedicine.

[54] Torres R., Toro L., Sanhueza M., Lorca E., Ortiz M., Pefaur J., Clavero R., Machuca E., Gonzalez F., Herrera P. (2022). Clinical Efficacy of SARS-CoV-2 Vaccination in Hemodialysis Patients. Kidney Int. Rep..

[55] Meijers R.W.J., Litjens N.H.R., de Wit E.A., Langerak A.W., Baan C.C., Betjes M.G.H. (2014). Uremia-associated immunological aging is stably imprinted in the T-cell system and not reversed by kidney transplantation. Transpl. Int..

[56] Kwok J.S.Y., Cheung S.K.F., Ho J.C.Y., Tang I.W.H., Chu P.W.K., Leung E.Y.S., Lee P.P.W., Cheuk D.K.L., Lee V., Ip P. (2020). Establishing Simultaneous T Cell Receptor Excision Circles (TREC) and K-Deleting Recombination Excision Circles (KREC) Quantification Assays and Laboratory Reference Intervals in Healthy Individuals of Different Age Groups in Hong Kong. Front. Immunol..

[57] Vagiotas L., Stangou M., Kasimatis E. (2022). The effect of Panel Reactive Antibodies on T cell immunity reinstatement following renal transplantation. World J. Transpl..

[58] Zhang Q.-Q., Xie Y.-L., Zhang W.-J., Wang F., Luo Y., Chen S., Chang S. (2021). Lymphocyte function based on IFN-γ secretion assay may be a promising indicator for assessing different immune status in renal transplant recipients. Clin. Chim. Acta.

[59] Elgueta S., Fuentes C., López M., Hernández J., Arenas A., Jiménez M., Gajardo J., Rodríguez H., Labraña C. (2011). Effect of Implementing Anti-HLA Antibody Detection by Luminex in the Kidney Transplant Program in Chile. Transplant. Proc..

[60] Guichard-Romero A., Marino-Vazquez L.A., Castelán N., López M., González-Tableros N., Arvizu A., De Santiago A., Alberú J., Morales-Buenrostro L.E. (2016). Impact of pretransplant exposure to allosensitization factors generating HLA antibodies in the Luminex era. Transpl. Immunol..

[61] Alelign T., Ahmed M.M., Bobosha K., Tadesse Y., Howe R., Petros B. (2018). Kidney Transplantation: The Challenge of Human Leukocyte Antigen and Its Therapeutic Strategies. J. Immunol. Res..

[62] Van Loon E., Lamarthée B., Barba T., Claes S., Coemans M., de Loor H., Emonds M.P., Koshy P., Kuypers D., Proost P. (2022). Circulating Donor-Specific Anti-HLA Antibodies Associate With Immune Activation Independent of Kidney Transplant Histopathological Findings. Front. Immunol..

[63] Velioğlu A., Aksu B., Asicioglu E., Arıkan H., Tinay I., Yardimci S., Yegen C., Tuğlular S., Özener C., Arikan H. (2015). Association of BK Virus Titers With Lymphocyte Count in Renal Transplant Patients. Transplant. Proc..

[64] Hoff U., Markmann D., Thurn-Valassina D., Nieminen-Kelhä M., Erlangga Z., Schmitz J., Bräsen J.H., Budde K., Melk A., Hegner B. (2022). The mTOR inhibitor Rapamycin protects from premature cellular senescence early after experimental kidney transplantation. PLoS ONE.

[65] Akalin E., Azzi Y., Bartash R., Seethamraju H., Parides M., Hemmige V., Ross M., Forest S., Goldstein Y.D., Ajaimy M. (2020). COVID-19 and Kidney Transplantation. N. Engl. J. Med..

[66] Elias M., Pievani D., Randoux C., Louis K., Denis B., DeLion A., Le Goff O., Antoine C., Greze C., Pillebout E. (2020). COVID-19 Infection in Kidney Transplant Recipients: Disease Incidence and Clinical Outcomes. J. Am. Soc. Nephrol..

[67] Kremer D., Pieters T.T., Verhaar M.C., Berger S.P., Bakker S.J.L., van Zuilen A.D., Joles J.A., Vernooij R.W.M., Balkom B.W.M. (2021). A systematic review and meta-analysis of COVID-19 in kidney transplant recipients: Lessons to be learned. Am. J. Transplant..

[68] Mahalingasivam V., Craik A., Tomlinson L.A., Ge L., Hou L., Wang Q., Yang K., Fogarty D.G., Keenan C. (2020). A Systematic Review of COVID-19 and Kidney Transplantation. Kidney Int. Rep..

[69] Stangou M.J., Fylaktou A., Ivanova-Shivarova M.I., Theodorou I. (2022). Editorial: Immunosenescence and Immunoexhaustion in Chronic Kidney Disease and Renal Transplantation. Front. Med..

[70] Imam A., Abukhalaf S.A., Imam R., Abu-Gazala S., Merhav H., Khalaileh A. (2020). Kidney Transplantation in the Times of COVID-19—A Literature Review. Ann. Transplant..

[71] Dębska-Ślizień A., Ślizień Z., Muchlado M., Kubanek A., Piotrowska M., Dąbrowska M., Tarasewicz A., Chamienia A., Biedunkiewicz B., Renke M. (2021). Predictors of Humoral Response to mRNA COVID19 Vaccines in Kidney Transplant Recipients: A Longitudinal Study—The COViNEPH Project. Vaccines.

[72] Alejo J.L., Mitchell J., Chiang T.P.-Y., Abedon A.T., Boyarsky B.J., Avery R.K., Tobian A.A., Levan M.L., Massie A.B., Garonzik-Wang J.M. (2021). Antibody Response to a Fourth Dose of a SARS-CoV-2 Vaccine in Solid Organ Transplant Recipients: A Case Series. Transplantation.

[73] Abedon A.T., Teles M.S., Alejo J.L., Kim J.D., Mitchell J., Chiang T.P.Y., Avery R.K., Tobian A.A.R., Levan M.L., Warren D.S. (2022). Improved Antibody Response After a Fifth Dose of a SARS-CoV-2 Vaccine in Solid Organ Transplant Recipients: A Case Series. Transplantation.

[74] Arora S., Kipp G., Bhanot N., Sureshkumar K.K. (2019). Vaccinations in kidney transplant recipients: Clearing the muddy waters. World J. Transplant..

[75] Moran J., Dean J., De Oliveira A., O’Connell M., Riordan M., Connell J., Awan A., Hall W.W., Hassan J. (2013). Increased levels of PD-1 expression on CD8 T cells in patients post-renal transplant irrespective of chronic high EBV viral load. Pediatr. Transplant..

[76] (2012). Guidelines for Vaccinating Kidney Dialysis Patients and Patients with Chronic Kidney Disease Summarized from Recommendations of the Advisory Committee on Immunization Practices (ACIP). https://www.cdc.gov/vaccines/pubs/dialysis-guide-2012.pdf.

[77] Kotton C.N. (2014). Immunization after kidney transplantation—what is necessary and what is safe?. Nat. Rev. Nephrol..

[78] Gopal B. (2016). Guidelines for vaccination in kidney transplant recipients. Indian J. Nephrol..

[79] Wang L., Verschuuren E., van Leer-Buter C., Bakker S., de Joode A., Westra J., Bos N.A. (2018). Herpes Zoster and Immunogenicity and Safety of Zoster Vaccines in Transplant Patients: A Narrative Review of the Literature. Front. Immunol..

[80] Caillard S., Thaunat O. (2021). COVID-19 vaccination in kidney transplant recipients. Nat. Rev. Nephrol..

[81] Demir E., Dheir H., Safak S., Artan A.S., Sipahi S., Turkmen A. (2022). Differences in clinical outcomes of COVID-19 among vaccinated and unvaccinated kidney transplant recipients. Vaccine.

[82] Bertrand D., Laurent C., Lemée V., Lebourg L., Hanoy M., Le Roy F., Nezam D., Pruteanu D., Grange S., de Nattes T. (2022). Efficacy of anti–SARS-CoV-2 monoclonal antibody prophylaxis and vaccination on the Omicron variant of COVID-19 in kidney transplant recipients. Kidney Int..

[83] Askari H., Sanadgol N., Azarnezhad A., Tajbakhsh A., Rafiei H., Safarpour A.R., Gheibihayat S.M., Raeis-Abdollahi E., Savardashtaki A., Ghanbariasad A. (2021). Kidney diseases and COVID-19 infection: Causes and effect, supportive therapeutics and nutritional perspectives. Heliyon.

